# Digital Commensality: Eating and Drinking in the Company of Technology

**DOI:** 10.3389/fpsyg.2019.02252

**Published:** 2019-10-09

**Authors:** Charles Spence, Maurizio Mancini, Gijs Huisman

**Affiliations:** ^1^Crossmodal Research Laboratory, Oxford University, Oxford, United Kingdom; ^2^School of Computer Science and IT, University College Cork, Cork, Ireland; ^3^Digital Society School, Amsterdam University of Applied Sciences, Amsterdam, Netherlands

**Keywords:** virtual commensality, digital dining, technology, Mukbang, social dining, solo dining, digital distraction

## Abstract

Commensality is a key aspect of social dining. However, previous research has identified a number of pros and cons associated with the incorporation of digital technology into eating and drinking episodes. For instance, those who are distracted by digital technology may eat/drink more (that is, they may overconsume) as a result of their failure to attend to the food-related sensations that are thought to cue the termination of eating. Similarly, it has often been suggested that the use of mobile devices at mealtimes can disrupt the more commensal aspects of dining/drinking (at least among those who are physically present together). At the same time, however, looking to the future, it seems clear that digital technologies also hold the promise of delivering opportunities for enhanced multisensory experiential dining. For instance, they might be used to match the auditory, visual, or audiovisual entertainment to the eating/drinking episode (e.g., think only about watching a Bollywood movie while eating a home-delivery Indian meal, say). Indeed, given the growing societal problems associated with people dining by themselves, there are a number of routes by which digital technologies may increasingly help to connect the solo diner with physically co-located, remote, or even virtual dining partners. In this review of the literature, our focus is specifically on the role of technology in inhibiting/facilitating the more pleasurable social aspects of dining, what one might call “digital commensality.” The focus is primarily on Westernized adults with reasonable access to, and familiarity with, digital technologies.

## Introduction

The term “commensality” refers to the positive social interactions that are associated with people eating together (see Sobal, 2000; see also Simnel, 1910/1994). Eating together is a hugely important social activity (e.g., see Spence, 2016, 2017a, Chapter 7), with evidence of communal feasting going back at least 12,000 years in the archeological record (Munro and Grosman, 2010). According to Jones (2008), feasting together is part of what sets us apart from many other species. As Camille Rumani, co-founder of the VizEat site, puts it a few years ago, it should never be forgotten that “The table is the original social network” (quoted in Spence, 2017a). Yet, the proliferation of the well-known digital social networks, all too often accessible at the dinner table through smartphones (Moser et al., 2016; Ferdous et al., 2016a,b), shows how current-day technology can impact commensality (in both a positive way and a negative way).

The stereotypical image is that technology exerts a negative influence on people’s experience of food and drink. One needs only to think of those individuals mindlessly eating in front of the television or of all those people currently eating together (i.e., at the same table) while seemingly distracted by whatever is going on in their digital feeds (i.e., on their mobile devices; see also Oldham-Cooper et al., 2011; Radesky et al., 2014). Indeed, the latter is now deemed a topic worthy of discussion in books on table manners; for example, see the newspaper column by Feiler (2010), on the acceptability of Googling at the dinner table. At the same time, however, there are also a number of potentially exciting opportunities offered by the incorporation of digital technology into/around mealtime activities, including novel technologies such as tele-dining (see Grevet et al., 2012).

**Figure 1:**
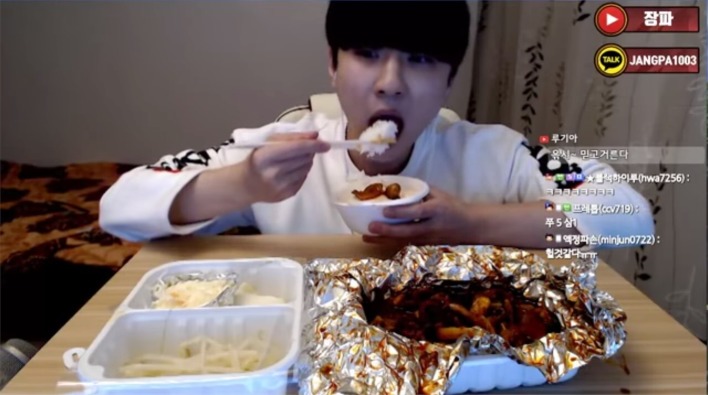
In what sense are those who engage in Mukbang involved in a meaningfully social/connected form of dining mediated by digital technology? There are purportedly huge numbers of young Koreans (Kim, 2018; Choe, 2019; though note that Mukbang is also gaining popularity amongst other Asian and Western viewers; Donnar, 2017; Pereira et al., 2019) eating alone while tuning in to a broadcast jockey who are normally reasonably attractive individuals seen eating large amounts of energy dense food (e.g., deep fried chicken wings). This figure shows a still image taken from Korean Mukbang channels (see Vice Food, 2015).

The term “digital commensality” is used here to cover a number of scenarios, from physically eating together with someone as a result of some digital technology-based intervention (as offered by the likes of websites such as VizEat; see Eleftheriou-Smith, 2017); Skeating – i.e., Skyping with a remotely located loved one, or friend, while eating (see Spence, 2017a), as well as more elaborate tele-dining installations that allow for some element of interactivity with those whom we may be dining with remotely (e.g., Wei et al., 2011); and Mukbang – eating by oneself at the same time as one watches someone else (a so-called broadcast jockey) eat alone over the Internet (Figure 1; see also Vice Food, 2015; Donnar, 2017; Kim, 2018; Choe, 2019; Pereira et al., 2019). While Mukbang first originated in Korea, it is interesting to note that the trend has now spread rapidly across other parts of Asia. The role of digital technology in mediating commensality also extends through to the seemingly more futuristic scenario, whereby one (the solo diner, that is) eats together with a digital agent (as in the case of assisted living robots for the elderly/infirm; see McColl and Nejat, 2013), or else, even more futuristic, with those who may be on a long-term mission to Mars (see Obrist et al., 2019; see also https://www.enib.fr/vrmars/index.html).

The focus of this review will be on the (digital) facilitation of commensality among the growing number of solo diners. However, as we will see below, the various digital solutions to alleviating the growing problem of solo dining may not all be equally feasible/effective in terms of delivering “digital” or “remote” commensality (Grevet et al., 2012). This article reviews the various ways in which digital technologies may historically have led not only to problems at mealtimes, but also (more importantly) to look at the various ways in which, in the future (and, in some cases, already), digital technologies may be offering a range of opportunities to enhance the experience of food [e.g., through the use of virtual reality (VR), augmented reality (AR), augmented tableware, projection mapping, sonic seasoning, etc.; see Narumi et al., 2012; Spence and Piqueras-Fiszman, 2013, 2014; Spence et al., 2016; Spence, 2017c]. We will not, however, be focusing on the role of digital technology in helping people to connect, or share, in the act of preparing/making food (e.g., Bell and Kaye, 2002; Mäkelä, 2009; Foley-Fisher et al., 2010). One might think here only about the phenomenal rise of digital cooking assistants such as Chef Steps^1^. Instead, our focus is squarely on the role of digital technology when consuming food and drink. It is important here to highlight the fact that our focus is primarily on westernized adults with good access to, and familiarity with, digital technologies, though, on occasion, we touch on work that deals with those at either end of the lifespan (i.e., children and older individuals). We end up outlining a number of suggested areas of future research concerning the more commensal aspects of dining, when some/all of the dining companion(s) are digitally mediated.

To provide a background against which to view digital commensality, we review diverse literatures on social dining practices. We will begin this review by providing an overview of available demographic data and research on solo dining and its most likely causes. We will outline potential negative consequences for health and well-being associated with solo dining regarding food intake and feelings of loneliness, as well as efforts that are being made to alleviate these negative aspects. We then turn our attention to the literature on the costs and benefits of dining together. Next, we consider how technology has the potential to be both a distractor in these commensal dining settings and offering opportunities to connect digitally enabled/savvy individuals in new ways. We conclude our review by considering the role that popular dining choices, such as food delivery services, may potentially have on digital commensality in the years ahead.

## Problems Associated With the Increase in Isolated Living

**Figure 2:**
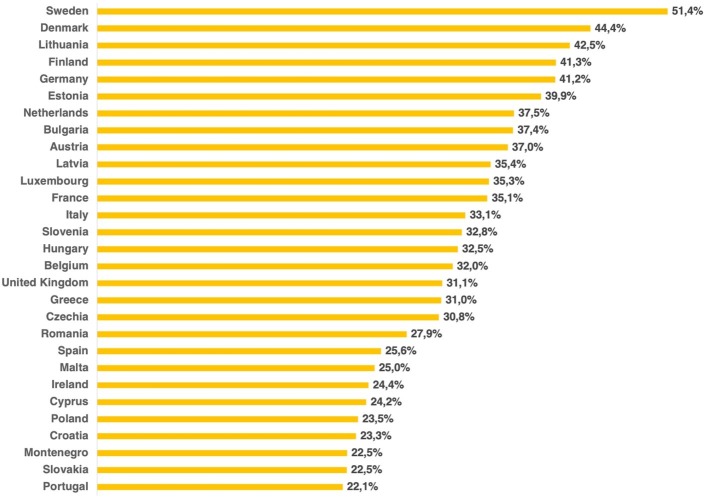
Single-person households as a percentage of the total number of households in 28 EU member states in 2017. Data from Eurostat (code lfst_hhnhtych).

The number of people living alone has increased steadily over recent decades (Gordon, 2017; see also US Census Bureau, 2018a,b). There has, for example, been a fivefold increase in the number of single-person households in the USA compared to the 1960s (US Census Bureau, 2018a,b). According to Euromonitor International, a leading market research firm, the number of people living alone is skyrocketing globally, rising from about 153 million in 1996 to 277 million in 2011 – that is, an increase of around 80% in 15 years. In the UK, 31% of households have one person living in them (see Figure 2), while in the USA, the figure is 28% (US Census Bureau, 2018b). According to Klinenberg (2012), the highest figure comes from Sweden, where 47% of households have only a single resident, though more recent data put that percentage at 51% (see Figure 2).

To give some sense of the problem in relation to food consumption, in Japan, where people live longer than in most other places, it has been estimated that 24% of pensioners eat the majority of their meals alone (see Tani et al., 2015a). Meanwhile, focusing now a little more specifically on the UK case (simply because more of the relevant data/research that we came across pertains to this group), a survey from 2016 conducted among 2000 UK adults indicated that “15% of respondents said they hadn’t had a meal with another family member in the last six months, 30% said they hadn’t done so with a best friend in the last six months, and 45% hadn’t done so with an old friend” (Dunbar, 2017, p. 201). Though exact data are scarce, according to anecdotal evidence, more people are eating a greater number of their meals by themselves (i.e., alone) than ever before (Klinenberg, 2012, 2013). Of course, it should be stressed that living alone is not in-and-of-itself necessarily an issue, but an increase in (chronic) loneliness as a result of living alone most certainly is (Hawkley and Cacioppo, 2010; Hurst, 2018). A Mintel survey in 2001, for example, found that three-quarters of British families had already abandoned regular meals, and 20% never sat down together to eat (quoted in Palmer, 2006, p. 34), while a *Grocery Retailing Report* from 2006 suggested that 51% of meals were eaten alone, as compared to 34% in 1994 (quoted in Steel, 2008, p. 339). Several reasons for this reduced commensality have been put forward over the years, including the growing aging population, increasing divorce rates, rising remote working (i.e., increasingly busy lifestyles; see also Statista Survey, 2016; Winsight Grocery Business, 2018), not to mention news reports of workers taking their lunch at their desk (see Muhammad, 2012). There has also been a rise in those reporting the need to eat on the run (see Statista, 2019b). Popular news media have described these developments with dramatic headlines such as “Death of the family meal” (Anonymous, 2015) and “The death of the dining table” (Anonymous, 2013). There are, of course, undoubtedly some important cultural differences here too, both around the proportion of the population who live alone in a given society and the acceptability/frequency with which people eat by themselves/eat out (see also Fischler, 2011). However, in order to put the issue of solo dining into some kind of context, it suffices to note that one in four Britons has no choice but to eat alone rather than having the luxury of partaking in a family meal, according to anecdotal media reports (see Anonymous, 2015).

This means that there is a very real danger that many of these single-person households may be missing out (knowingly or otherwise) on the benefits of commensality. This is especially worrying given the suggestion that “eating alone is the most extreme form of feeling disconnected in our culture” (Van Goor, quoted in Balfour, 2014). According to Sobal and Nelson (2003, p. 182), “Eating alone is devalued and is not considered a ‘real’ meal for many people” and furthermore, “almost all people (who were surveyed) thought that an ideal meal should be eaten with the company of others.” In fact, a large body of empirical research has convincingly demonstrated that social dining typically has beneficial effects on both a diner’s nutritional status and their social/emotional well-being (see Fulkerson et al., 2014, for a review).

The break-down of the nuclear family (related to the preceding points) has also been blamed for the increase in solo dining. That said, some have questioned whether there really ever was such a thing as a nuclear family dinner, or whether instead that is nothing more than a contemporary middle-class conceit (e.g., Murcott, 1997; Steel, 2008, on this point; see also Mestdag, 2005). Certainly, the rose-tinted view that family meals were commonplace in the past may be something of a myth, or at least only true at certain points in our history (see also Laurier and Wiggins, 2011). According to some commentators, families may not actually have been dining as a unit here in the UK a century ago (nor presumably in many other industrialized countries either). Rather, it has been suggested that the mother would eat with her children, and later, when the breadwinner came home from work, he would probably have consumed the meal that had been prepared by his wife, eating alone and likely without conversing (see Johnston, 1977, p. 13; see also Douglas and Nicod, 1974; Rotenberg, 1981).

As far as the more social aspects of dining are concerned, it is important to recognize that not all dining companions are equivalent in terms of the effect (either positive or negative) that they (may) exert over a diner’s physical and mental well-being (e.g., see Salvy et al., 2007; Young et al., 2009; Cruwys et al., 2012, 2015). Here, it should be noted that we are not simply contrasting human versus digital/virtual dining companions but distinguishing between whether one happens to be dining with friends, family, work colleagues, acquaintances, or else with unknown strangers or digital agents. The evidence concerning the beneficial effects of being a part of family meals on a range of dependent measures is striking and includes positive outcomes in terms of both health/weight and social development (e.g., Neumark-Sztainer et al., 2003; Delistraty, 2014; Fulkerson et al., 2014; Goldfarb et al., 2014; Dunbar, 2017). Indeed, the benefits of sharing family meals together tend to be especially pronounced among children/adolescents (e.g., Coon et al., 2001; Hammons and Fiese, 2011; though see also Ochs et al., 1996; McIntosh, 1999; Ochs and Shohet, 2006; Fitzpatrick et al., 2007; Sobal and Hanson, 2011).

The key point to stress here is that there are a number of well-documented negative health consequences associated with living/eating alone (e.g., Marshall et al., 1999; Quigley et al., 2008). Negative consequences exist both in terms of food consumption (with undereating being documented at one extreme and overeating at the other; e.g., Tani et al., 2015a) and in terms of depressed mood and a loss of social connectivity (i.e., leading to a decreased feeling of well-being; e.g., Conklin et al., 2014; Tani et al., 2015b). There are also potential cost/waste implications associated with solo dining/living: So, for instance, according to one study by the Waste and Resource Action Program, those UK residents who live alone tend to throw away roughly £290 of food and drink per year, £90 more than those living with others (Quested and Luzecka, 2014). The figures are presumably likely to be similar in other countries too.

### On the Costs and Benefits of Dining Together

Social dining does not in-and-of-itself guarantee better eating behaviors for the individual(s) concerned. Rather, the research shows that there may be dangers associated with dining with too many other people. In particular, a number of studies conducted over the last quarter of a century or so have demonstrated that the amount of food that people consume can be described by a power relation with the number of people dining together (e.g., de Castro et al., 1990; de Castro and Brewer, 1992; Clendenen et al., 1994; Bell and Pliner, 2003; Hetherington et al., 2006; see Herman et al., 2003; Herman, 2015, for reviews). In fact, according to Herman (2017), a primary reason for social eating may actually be that it provides an opportunity for people to overindulge. Though, that being said, a number of other factors have been shown to modulate the increased consumption that is typically seen in group settings (e.g., de Castro, 1990, 1994; Goldman et al., 1991; Feunekes et al., 1995; Klesges et al., 2006; Cavazza et al., 2011; Higgs and Thomas, 2016).

At the same time, however, commensality may have multiple beneficial effects on diners (see Grimes and Harper, 2008), including the positive mood/emotion likely engendered by eating with others (rather than eating alone; Troisi et al., 2015). However, beyond that, a separate literature shows that shared experiences seem to be amplified (Boothby et al., 2014). For instance, Boothby and colleagues conducted research specifically in the context of shared food experiences. Across two studies, the authors found that when shared with someone else, pleasant food (i.e., chocolate) tasted better, while unpleasant food was rated as tasting worse.

Questionnaire research has revealed that people like to converse while dining: For instance, according to one survey, only 3.7% of the 244 US adults questioned preferred to eat in silence, while 58.8% preferred to eat while conversing with others. Meanwhile, a further 6.2% of those quizzed preferred to eat while listening to music (Pellegrino et al., 2015). Similarly, Larson et al. (2009) also reported that younger adults preferred to eat with others, despite the fact that they reported not always having the time to do so (see also Poulain, 2002). What is more, according to the research, we are more likely to trust a stranger who eats the same food as us (Woolley and Fishbach, 2017). In summary, therefore, there are both potential benefits and costs to commensal dining. However, on balance, it can be argued that the benefits would seem to outweigh the costs when compared to enforced solo dining (e.g., due to enforced isolated dining among the growing number of elderly people living alone).

### Eating Out Alone – Losing Its Stigma

Of course, living alone need not necessarily mean eating alone. After all, some proportions of people’s meals are likely eaten outside the home environment. According to Steel (2008), that figure was around a third of meals (eaten outside of the home) here in the UK. Indeed, a survey conducted in 2016 indicated that 36% of Britons eat out once or twice a week (see Food Standards Agency, 2016; Statista, 2016). According to Steel, this figure was likely to be somewhere closer to 50% in the USA, although note that one recent large-scale survey (conducted in July 2018) has actually put this figure at much closer to 20% (NPD Group, 2018). Estimates on this question do seem to vary widely from one report to the next (see the news report by Ferdman, 2015). That said, one of the problems traditionally in the UK has always been that many people have tended to feel self-conscious about eating out by themselves (the worry being that they would look like lonely losers to anyone who caught sight of them; e.g., Jonsson and Pipping Ekström, 2009; Pliner and Bell, 2009; Danesi, 2012; c.f., Ratner and Hamilton, 2015). Increasingly, however, solo dining seems to be losing its stigma (at least in those parts of the world where people felt that there was a stigma attached in the first place). In part, as suggested in a news article by Freedom du Lac (2011), this could be because mobile devices (i.e., a form of digital technology) now enable many more solo diners to distract themselves and/or perhaps socialize with other people while being physically alone at the table (see also the news report by Luckhurst, 2015).

In recent years, the rise in the number of people dining alone (see OpenTable, 2015) has started to attract the attention of both the press and the restaurateurs (e.g., see Victor, 2015), with a growing number of commentators, perhaps for the first time, starting to promote the merits of solo dining (e.g., see news reports by Balfour, 2014; Muston, 2015; Frizzell, 2016; Levine, 2016). While it may still only represent a small percentage of total reservations, sites like OpenTable (an online reservation service) reported an 80% increase between 2014 and 2018 (Pavia, 2019). That said, it is important to note that deliberately choosing (on occasion) to dine out alone (e.g., as when the chef, or food critic, wishes to concentrate on the food that s/he is eating) is a completely different situation from having no alternative but to eat alone, which as we saw earlier, is the situation for a growing number of individuals. According to Gordon (2017): “For the elderly, being single is not a choice. As life expectancy rises and the number of elderly people swells, there is a growing number of widowed, divorced or otherwise single homes populated by persons aged 65+. This is accelerating as the extended family unit is being broken up.” Nevertheless, whatever the historical situation once was, at present, it is clear that the benefits of eating together are especially pronounced among the elderly (Wright et al., 2006; see also Torres et al., 1992; de Castro, 2002; Spence, 2017b).

**Figure 3:**
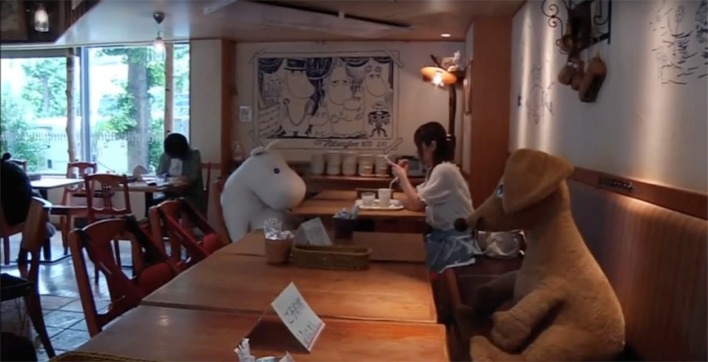
Solo diners eating with a cuddly toy. A new trend in tackling loneliness from one Japanese restaurant (figure reprinted from Fishwick, 2014).

On the positive side (from the perspective of the solo diner), a number of restaurants/chains have started to offer dining solutions that are less awkward for them: Everything from the introduction of the chef’s table (or think of eating/drinking at the bar as is common in North American bars/restaurants) through to dining alone together with stuffed animals in one Japanese restaurant (see Figure 3; Fishwick, 2014). Elsewhere, restaurant chains such as *Wagamama* have made a great success of putting separate (groups of) diners together on the same long tables. This novel approach challenges the standard schema (at least traditionally in UK restaurants) of each group of diners eating at their own table (see Spang, 2000, on the history of the restaurant, and its peculiar social arrangements), though there is a legitimate question here concerning what exactly counts as social dining. In other words, is it enough simply to be at the table with other people, or do they need to be part of one’s own group (see Hirsch and Kramer, 1993)? Sharing the table, or counter, is, of course, standard practice in many countries (e.g., at sushi restaurants in Japan, or when eating tapas in Spain or Pintxos in the Basque country). It can be argued that the rise of the chef’s table (while undoubtedly adding a dash of theatricality to proceedings) can also be framed in terms of providing a means of enabling single diners to enjoy a meal out without having to feel uncomfortable about eating alone. There may, though, be cultural differences in the acceptability and/or occurrence of such communal dining practices in those from different cultures (Fischler, 2011; see also Armstrong, 2009, for anecdotal evidence specific to British culture).

A more explicit statement about solo dining has been made by the proposed *Eenmaal* chain of restaurants in the Netherlands, where there are only tables for one (e.g., Sanghani, 2014)^2^. Note that dining at *Eenmaal* does not seem to be about stopping by for a bite to eat, but *rather* actually making a statement by deliberately choosing to eat alone with other solo diners. Separately, there are also projects such as the Eden Project’s Big Lunch^3^, which has been running for a few years here in the UK, with the aim being to get as many people as possible to eat with their neighbors on at least 1 day each year. At the same time, in recent years, a number of websites have emerged that help to connect diners, no matter whether or not they are solo. VizEat being one successful example of this approach (e.g., see Eleftheriou-Smith, 2017). While VizEat started out in the UK, several similar sites have since sprung up across the globe, their stated aim, to connect people whenever they happen to be, even when traveling in a foreign country, say (see Rumbelow, 2015). In North America, for instance, the equivalent site is called EatWith^4^.

## Technology Distracts/Separates

The traditional way in which technology interfered with dining would have been in terms of the so-called TV dinner (e.g., see Lanza, 2004). According to Gore et al. (2003), almost half of all weekly meals are reportedly consumed in a room with a television switched on. An extensive body of research has demonstrated the negative influence on consumption, meaning that distracted dining (e.g., while watching the TV) leads to increased consumption (e.g., Bellisle and Dalix, 2001; Blass et al., 2006; Boulos et al., 2012; Braude and Stevenson, 2014; see also Fitzpatrick et al., 2007). The magnitude of the increase in consumption that is seen when people dine in front of the TV depends on the kind of show that the diner happens to be watching (Chapman et al., 2014), not to mention whether it is a repeat (Mathur and Stevenson, 2015). In the worst case scenario, people have been shown to eat a third more food with the TV on. Importantly, these effects seem to occur independently of any potential influence of food advertisements on food intake during TV viewing and are, in fact, not limited to television but apply to screen-based devices (e.g., smartphones) in general (Marsh et al., 2013)^5^.

The potential dangers associated with overeating (e.g., as a result of distracted dining) come to the fore when one considers that according to estimates published 5 years ago, nearly 70% of adults in the USA are overweight and close to 40% are considered obese (see National Center for Health Statistics, 2014; see also Lifshitz and Lifshitz, 2014). One needs only to examine the increasing number of screen-based devices, such as smartphones, laptops, and tablets in order to see how current technological developments might fuel the negative effects of screen time on food intake. According to the results of the latest research, using a smartphone at mealtimes results in a significant increase in caloric ingestion (da Gonçalves et al., 2019). In a lab-based study, conducted during several consecutive days, the authors found that calorie intake increased for both smartphone use and reading a printed text as compared to a no-distraction baseline. It would thus seem that any distractor can potentially impact calorie intake, but one could argue that smartphones provide a particularly tempting, not to mention readily available, form of distraction. Indeed, other research has shown that increased screen time, including time using smartphones, playing video games, and watching television, is associated with a higher body mass index (BMI) in adolescents (Cameron et al., 2016). This relation was mediated by calorie intake, in particular the intake of carbohydrates. The authors suggest that reducing screen time may reduce caloric intake and thus help weight management in obese adolescents.

Finally, other researchers considered the impact of using multiple screens at the same time on snack consumption (e.g., watching TV while using one’s smartphone to send a text) and found that participants consumed significantly more unhealthy compared to healthy snacks in the experimental condition where they were tasked with watching TV, texting, and reading text online (Kononova et al., 2018). Note, however, that conclusive evidence regarding the influence of the presence of multiple screens on food consumption remains to be documented (Marsh et al., 2015). Regarding the distracting influence of screen time, in particular smartphone use, during food consumption and its effects on calorie intake, it is worth considering that solo diners might be more inclined to make use of distractors while eating alone. In this situation, smartphones may both provide a distraction and engender a feeling of connection to others (e.g., by providing access to social media applications; see also Phua et al., 2017) with potentially negative consequences on food intake, as outlined above.

Of course, technology can also directly interfere with the more commensal aspects of dining. Just think, for instance, of the increasingly common scene of people sitting together at a dining table in a restaurant, but with each one staring into their own mobile screen (e.g., O’Hara et al., 2012; Hiniker et al., 2016; Ferdous et al., 2016a,b). This a trend that some restaurateurs have expressed their displeasure about (see Ensor, 2013). People who are physically together, but seemingly isolated, at one and the same time (see also Rimer, 2009 for a report on families’ struggles in this regard), which may, in fact, have a negative impact on their perception of face-to-face social interactions (Rotondi et al., 2017), including those at the dining table. Taking a picture has been shown to enhance people’s memory for what they ate, even if they do not look at that picture again (Coary and Poor, 2016). At the same time, however, it is important to note that excessive media use has been shown to impair people’s memory for various kinds of experience (Tamir et al., 2018; see also Robinson et al., 2013). Dining is likely to be just like other kinds of experience in this regard.

It is currently something of an open question as to whether it matters exactly whom one is conversing with at the table – i.e., with someone who is physically present, or else remotely connected by one’s mobile screen. Who knows, perhaps virtual, or rather digital, dining companions are as good as the real thing? Only future research will tell.

## Technology Connects

Some intriguing early scoping research addressed the question of how to connect diners who wanted to share a meal while physically separated, perhaps by a very long distance (see Wei et al., 2011; Barden et al., 2012; Heidrich et al., 2012; Nawahdah and Inoue, 2013; Comber et al., 2014, 2015). Grevet et al. (2012) refer to this as “remote commensality.” The question, in this case, was whether technology could be used to facilitate the connection between those who are physically separated (see Ferdous et al., 2016a,b; Ferdous et al., 2017). Exploring social presence and connectedness at the telematic dinner party was one of the themes of this ground-breaking early work. One of the intriguing solutions explored in this context involved techniques for connecting diners (sometimes referred to as tele-dining), such as by giving both parties access to some form of shared food interaction. Below, we briefly explore a number of different instantiations of digital technology that offer various kinds (or levels) of digital commensality.

### Mukbang

This Korean term refers to the increasingly common habit among millions of predominantly Asian consumers (although it should be noted that there is also growing interest among Western audiences as well; see Donnar, 2017; Pereira et al., 2019) who live and eat at home alone to tune in to a broadcast jockey over the Internet at mealtimes (Kim, 2018; Choe, 2019; Pereira et al., 2019; see also Vice Food, 2015). Because of the large portions of energy dense foods that the latter are normally seen eating (Figure 1; Donnar, 2017), Mukbang undoubtedly raises some intriguing questions concerning whether people’s consumption behavior is influenced, potentially in a detrimental manner, by the person seen eating (c.f., Seddon and Berry, 1996; Pliner and Mann, 2004; Strahan et al., 2007)^6^.

One fear is that our consumption norms may be set by what we see others consume. Hence, if we see a person consuming a large energy-dense meal, it may turn out that we are “nudged” to consume more than we otherwise might (see Spence et al., 2016, on this theme). Indeed, in a study conducted among young women, Hermans et al. (2009) found that participants ate more when they observed a peer consuming more food, though only in a context in which the experimental confederate did not engage in social interaction with the participant. One might think that this scenario comes very close to that of passively watching a Mukbang video while eating (though note that during live streaming versions of Mukbang text chat with the broadcaster is possible; see Choe, 2019). Meanwhile, other research suggests that similar effects on social eating behavior can be explained by social comparison (Polivy and Pliner, 2015; Polivy, 2017) or mimicry (Hermans et al., 2012). These may be influenced by the food that is selected and body-type of the person observed (McFerran et al., 2009).

Similar effects already occur when one simply observes images of others eating, at least as far as taste perception is concerned (Poor et al., 2013). According to the latter researchers, seeing an image of another individual eating unhealthy food can all too easily be taken as social proof that indulging in unhealthy foods is both acceptable and appropriate. However, separate from Mukbang’s possible effect on consumption behavior, one might also want to question whether this kind of dining (with a digitally present ‘dining companion’) provides any kind of social benefits. That is, does any actual social interaction even need to take place for the benefits of eating together to be observed? Is Mukbang sufficient? This discussion raises a number of interesting contrast cases that are worth pausing to consider (see also Zhou et al., 2017).

What about eating in front of a mirror, for example (see Spence, 2018)? Is synchronized eating activity sufficient?^7^ Intuition says that this cannot be enough, but robust experimental data are undoubtedly needed to be sure. There are also intriguing questions here about the impact of dining in front of a mirror – again, this is a situation in which a person eats while there visually appears to be someone present (Nakata and Kawai, 2017). In a short-term study, Nakata and Kawai demonstrated that both young and elderly participants consumed a little more popcorn when eating in front of a mirror (or rather a screen showing themselves from the waist up) and rated the food as tasting better than when the screen showed a blank wall^8^. Given its huge popularity in parts of Asia, further research is clearly (one might say urgently) needed in order to get a better sense of the potential costs/benefits of Mukbang. According to the scarce empirical research, primary reasons for people (in both Asian and Western cultures) to watch Mukbang is due to the physical attractiveness of the host and because of social normative influences (Pereira et al., 2019). No significant effects were found for feelings of loneliness (e.g., statements such as “I lack companionship”; Pereira et al., 2019, p. 85) in the decision to watch Mukbang. As yet, it would seem fair to say that it remains unclear whether or not it counts as a meaningful example of digital commensality, our guess is that it probably does not.

### Artificial Dining Assistants

**Figure 4:**
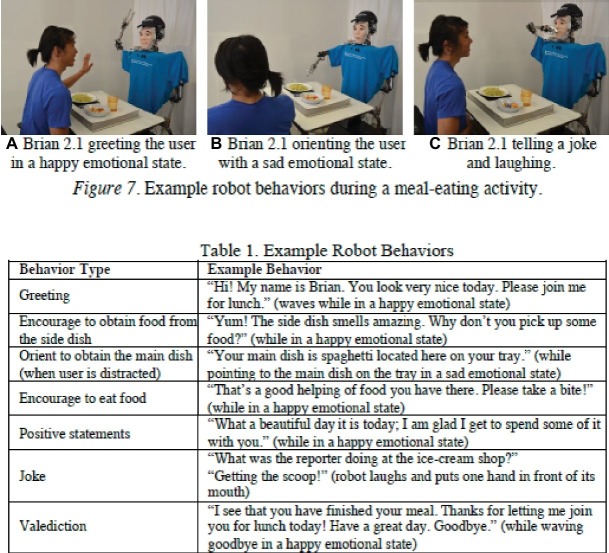
Assisted living robot – stills from interaction and example behaviors – specifically example robot behaviors during a meal-time encounter. [Reproduced from McColl and Nejat (2013), Figure 7 and Table 1 under the Creative Commons Attribution license].

One way to capitalize on the potential benefits that social dining through technology, such as Mukbang, might provide is by creating purpose-built artificial dining assistants (e.g., McColl and Nejat, 2013). Given the growing number of elderly individuals (either in care or in other assisted living situations), there are huge concerns/needs around assisted eating (Schell and Kayser-Jones, 1999). Indeed, failure to eat among this age-group is a well-recognized problem. Socially assistive robots such as Brian 2.1 (see Figure 4) offer one potential solution to the problem, as well as raising some intriguing questions about the degree/quality of commensality with a digital dining companion whose “job” it is to assist/guide eating. Positive preliminary data were obtained in one small-scale study with eight elderly care residents (all over 80 years of age). The robot was reported to have a beneficial effect as far as a number of the patients reporting positively on the interaction. Of course, one would want to see much larger-scale research and in the long term (who knows, Brian 2.1’s repertoire of jokes might become stale after a while!).

Elsewhere in the world of artificial assistants, one finds Ritschel et al. (2018) discussing a robotic drinking coach. Sometimes dining assistants can assume the aspect of kitchen objects, as in the case of Hermsen et al. (2016) testing an intelligent fork that provides haptic feedback when detecting a fast pace of eating (so people will become more aware of their eating habits, as demonstrated in the paper; though Hermans et al., 2017, found that such vibrotactile-augmented cutlery did not have a beneficial effect on reducing consumption). Meanwhile, Randall et al. (2018) created Health-e-Eater, a magic plate and a robotic companion, which motivates and educates children during meals. In such cases, note, the extent to which a diner’s behavior is influenced by that of a digital avatar or a robot (as in the phenomenon of imitation) is likely to be mediated by the sense of presence (see Fox et al., 2009).

**Figure 5:**
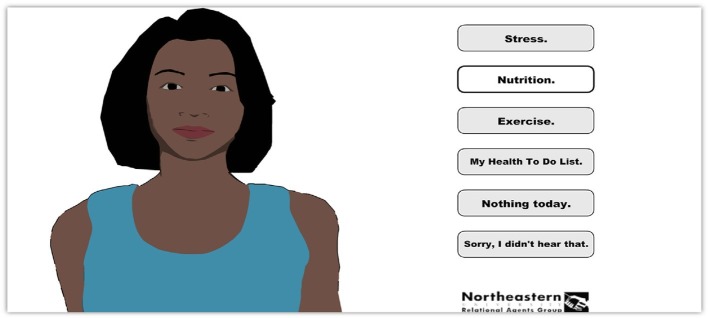
Gabby, the Embodied Conversational Agent (ECA) interface used in a randomized control trial demonstrating increased consumption of fruit (2 portions a day on average) when compared to patient education sheets. This ECA supposedly simulates face-to-face interaction (figure reprinted from Gardiner et al., 2017).

A more elaborate randomized controlled trial study was conducted by Gardiner et al. (2017). This study involved 61 women living in urban environments interacting with Gabby (see Figure 5), an embodied conversational agent (i.e., a virtual character). Gabby provided coaching on how to adopt a more healthy lifestyle, such as encouraging people to consume more fruit, stress management, mindfulness, and physical exercise. The results revealed a twofold increase in fruit consumption when compared to the experimental control. In line with this finding, Baroni et al. (2014) demonstrated that a humanoid robot (called Nao) could be used effectively to encourage children (*N* = 80) to eat more fruits and vegetables. However, note that embodied conversational agents such as Gabby are more coach than necessarily commensal dining companion. Meanwhile, Parra et al. (2018) described Lucy, a digital assistant designed to monitor people’s eating behaviors in order to help them to lose weight. An individual who needs targeted assistance with eating might also expect to develop some kind of relationship with a digital food assistant. It is currently unclear, though, how far along this path Brian 2.1, Nao, Gabby, or their successors currently are. The aim here, for those developing many of these applications, is to promote meal-time independence (Osborn and Marshall, 1992). However, it should be noted that simply ensuring adequate nutritional intake, while important, does not in-and-of-itself guarantee commensality (at least for those geriatric patients still capable of meaningful commensal interactions).

### Skeating

**Figure 6:**
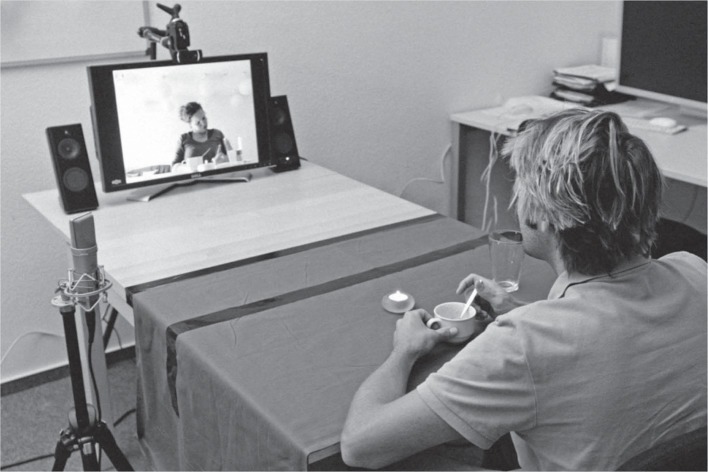
RoomXT, advanced video communication for joint dining over a distance from Heidrich et al. (2012) (figure from Heidrich et al., 2012).

Skyping while eating could potentially provide the benefits of co-dining for individuals in different geographical locations. Indeed, researchers have developed several systems that allow remote diners to share their mealtime activities. Systems such as KIZUNA (Nawahdah and Inoue, 2013) enable asynchronous dining interactions between people living in different time zones. RoomXT provides another solution for synchronous (or spontaneous) dining at a distance, with trompe l’oeil used to visually extend the dining table into the virtual environment (Figure 6; Heidrich et al., 2012). However, it is important to stress that merely watching a pre-recording of a person dining, as happens, for example, with the CU-Later system (Tsujita et al., 2010), is simply not going to be enough to have the illusion of co-dining, as fundamental non-verbal communicative cues, such as synchronization between the actions of “co-diners,” are missing, a problem that is further underlined in the design of the CoDine system (Wei et al., 2011). Could there be benefits in future remote dining systems that are able to provide an enhanced sense of commensality between remote dining partners? Would it be confusing if the various parties happen to be in different time zones? Would the commensal benefits be greater if both parties are eating at the same time, or does that not matter? As the reader can probably tell by this stage, there are a number of important questions awaiting an empirical answer in this space^9^. It might be that matching the background music in both venues would provide a contextual means of connecting people. For example, the tempo of the background music would appear to be directly connected to eating (Roballey et al., 1985; Caldwell and Hibbert, 2002) and drinking (McElrea and Standing, 1992) speed (see Spence et al., 2019, for a recent review). Once again, it is a case of more research needed.

In summary, the three broad approaches to digital commensality reviewed in this section, namely, Mukbang, artificial dining assistants, and Skeating are all potentially promising. And while there are challenges associated specifically with each of these approaches, taken as a whole the primary criticism to date must be in terms of the limited extent of research investigating the extent to which these approaches deliver the same health/well-being benefits that are associated with physically dining together with another person/other people. What is more, the sample sizes in the research that have been conducted to date tend to be rather on the low side, hence raising the possibility that many of the studies may be underpowered. Finally, and as highlighted by one of the original reviewers of this article, it still feels as though the field of digital commensality research lacks sufficient clear demonstrations that eating with technology alone or in a group is better than eating without technology. What is more, once the basic observations have been confirmed by suitably powered studies, future research will then need to focus on determining the psychological mechanisms underlying such beneficial effects on people.

## Conclusions

As this review of the literature has hopefully made clear, there are both current problems and a number of future opportunities associated with the merging of digital technology and eating/drinking. On the negative side, there is robust evidence to suggest that technology can (and/or is currently) potentially distracting us from our food as well as from the company we are physically with. This kind of mindless eating has been shown to result in increased consumption (e.g., see Robinson et al., 2013) and a lack of interaction with those whom we are physically dining with (see Fitzpatrick et al., 2007). At the same time, however, it is also clear that digital technology holds the potential to enhance both our experience of the food and the more social aspects of dining (see also Grevet et al., 2012). Several approaches to using technology to connect those who, for whatever reason, find themselves alone have been discussed and include Mukbang, artificial dining assistants, and Skeating. While all three approaches look potentially promising, as made clear in the preceding section, further suitably powered research is needed before any strong conclusions can be drawn concerning the merits, in terms of health and well-being of these digital commensality solutions.

As noted in the Introduction, the focus of this review has primarily been on the topic of “digital commensality” among adults. Equally important, of course, is to consider the same themes/issues as they pertain to other groups, such as children and older individuals. It is worth noting that at several points in the text we have come across the suggestion that the social aspects of dining are particularly important among those individuals at either end of the age spectrum. Additionally, in the future, it is obviously going to be important to consider how digital commensality operates between different generational groups. While currently in the West, an individual’s familiarity with those technologies that are relevant to the theme of digital commensality likely declines with increasing age, the situation is likely to change as the pre-digital consumers inevitably die-out. However, while these issues are undoubtedly important, the most that we can do here, given the paucity of empirical data that we have been able to find, is to flag these issues up as important topics for future research. Intergenerational friction when adopting future digital commensality solutions should certainly be borne in mind by those working in this area in the future. Further broadening the challenges associated with dealing satisfactorily with the topic of digital commensality is the question of how to deal with different cultural norms (e.g., in different parts of the world). Again, adequately detailing/dealing with cultural differences lies beyond the scope of the present article. The reader should nevertheless be aware that the majority of the examples discussed here deal specifically with digital commensality in the UK/North America, and in parts of East Asia (e.g., Korea, Japan). As such, only future research will reveal whether the same conclusions can be drawn when considering the opportunities and challenges around introducing digital commensality in other parts of the world.

### Future Solutions to Enhancing Digital Commensality Through Food Delivery Services

Another potentially interesting future development to tap into is home food delivery, which is becoming ubiquitous in a number of Western urban areas (just think Google Munchery, UberEats, Deliveroo, JustEat, etc.; see Statista, 2017, 2018, 2019a). A growing number of companies are now providing consumers with their food, raw, part-prepared, or ready-to-eat *via* their technology (e.g., Blue Apron, see Severson, 2016; HelloFresh; see Pesce, 2016). One obvious question here is that if a company such as UberEats, say, knows that it is sending out a meal for one, then why not offer to connect that solo diner with another solo diner?^10^ Not only this would appear to be a great opportunity (and should add value for the food provider too, given that their customers are likely to enjoy their meal experience more), but this also raises a number of questions, to which there is not, as yet, an empirical answer. Put simply, what are the minimal conditions for social dining/commensality? Is social dining beneficial even if one does not know the other person/people involved? Is commensality enhanced if two people, who are remotely dining together, eat the same food? Or, given that we often order different meals even when physically dining together, perhaps the same style of cuisine is sufficient. Or does the nature of the food itself not matter?^11^

While there are a number of potential situations in which digital technology can potentially be used to facilitate eating/drinking, the one that we have been most interested in here is the use of technology to facilitate commensality (i.e., the more social aspects of the interaction). This suggestion builds on the notion that social interaction at mealtimes is likely to have both a beneficial effect on mood, emotion, and/or well-being of those who, for whatever reason, might otherwise happen to be dining solo (many with increasing regularity). Digital technology can undoubtedly be used to connect groups of individuals who happen to be separated physically, be they at home or while away (e.g., on holiday; see Rumbelow, 2015). Alternatively, however, digital technology can also be used to offer other kinds on “social” interaction, with digital avatars or robot assistants (McColl and Nejat, 2013). Mukbang (Donnar, 2017; Kim, 2018; Choe, 2019; Pereira et al., 2019; see also Vice Food, 2015) offers another intriguing kind of food-related interaction with another person (albeit one who is not physically present). However, as this review has made clear, there are many important outstanding questions, with regard to the quality and type of commensal relation/interaction it may be possible to have in the future where digital commensality (e.g., with embodied conversational agents) is ever-more common (see also Horowitz, 2010; Marx, 2018).

## Author Contributions

All three authors assisted in the writing and editing of this review. Each author contributed to making comments on several drafts of the manuscript.

### Conflict of Interest

The authors declare that the research was conducted in the absence of any commercial or financial relationships that could be construed as a potential conflict of interest.

## Footnotes

^1^https://www.chefsteps.com/

^2^Somewhat peculiarly, though, many of the promotional materials for this dining concept show an empty restaurant.

^3^https://www.edenprojectcommunities.com/thebiglunchhomepage

^4^https://www.eatwith.com/

^5^Looking to the future, coordinating any audiovisual entertainment so that it actually matches or enhances the food/drink is one direction in which technology may come to enhance, rather than distract, from our eating and drinking (see Spence, 2017a).

^6^Though it should be noted that Wansink’s work has been brought into question in recent years (e.g., see van der Zee et al., 2017; http://www.brianwansink.com/phd-advice/statistical-heartburn-and-long-term-lessons).

^7^While there is clear evidence of turn-taking in conversation (e.g., see Goodwin, 1981), there is less evidence, in the case of dining, and interleaving dining with talking (Mondada, 2009). Note also the delays that are sometimes introduced when communicating over the Internet will likely also apply to dining. It is an open question as to how such temporal desynchronization affects the experience of sharing a meal.

^8^It is, though, worth stressing that the results of a number of other published studies have shown that whether eating in front of a mirror leads to a change in people’s choices/consumption behavior depends both on the food that they happen to be consuming (e.g., whether it is perceived as healthy or not) and on whether the person (seen in the mirror) happens to is obese, overweight, and/or concerned about their weight or not (see Spence, 2018, for a review).

^9^And finally, while increasing the social elements at mealtimes is generally seen as a good thing, it is important to note that there is also a negative side to such interactions – think here only of the anorexia sites (see Spence et al., 2016; Spence, 2017a, on this theme).

^10^Here, it is worth noting how KLM recently started offering its solo Business Class passengers, with their permission, to be seated together on their long-haul flights with other like-minded individuals (see Spence, 2017a).

^11^There may also be the opportunity to synchronize the entertainment with what is consumed (while not strictly commensality, this may nevertheless be a product-extrinsic digital meal enhancement opportunity; see also King et al., 2004). Indeed, there have already been a number of marketing-led interventions in this direction (Spence, 2017a).

## References

[ref1] Anonymous (2013). The death of the dining table: Most of us only sit down for a meal at the table a handful of times each YEAR. *Daily Mail Online*, April 29th. Available at: https://www.dailymail.co.uk/femail/article-2316515/The-death-dining-table-Research-shows-sit-meal-table-handful-times-YEAR.html (Accessed September 26, 2019).

[ref2] Anonymous (2015). Death of the family meal as one in four eat alone: Skipping dinner also increasingly common as our busy lifestyles take over. *Daily Mail Online*, September 29th. Available at: http://www.dailymail.co.uk/news/article-3252811/Death-family-meal-one-four-eat-alone.html (Accessed September 26, 2019).

[ref3] Armstrong H. (2009). Sharing tables with strangers: Do we British have a problem with sharing? *The Guardian*, September 23rd. Available at: https://www.theguardian.com/lifeandstyle/wordofmouth/2009/sep/23/sharing-table-restaurants (Accessed September 26, 2019).

[ref4] Balfour B. (2014). Tables for one – the rise of solo dining. *BBC News Online* Available at: http://www.bbc.co.uk/news/business-28292651 (Accessed September 26, 2019).

[ref5] Barden P. Comber R. Green D. Jackson D. Ladha C. Bartindale T. (2012). “Telematic dinner party: designing for togetherness through play and performance” in *Proceedings of the Design Interaction Systems Conference* (DIS ‘12). New York, NY: ACM Press, 38–47.

[ref6] Baroni I. Nalin M. Zelati M. C. Oleari E. Sanna A. (2014). “Designing motivational robot: how robots might motivate children to eat fruits and vegetables” in Robot and human interactive communication, 2014 RO-MAN: The 23rd IEEE international symposium on robot and human interactive communication (Edinburgh, UK: IEEE), 796–801.

[ref7] Bell G. Kaye J. (2002). Designing technology for domestic spaces: a kitchen manifesto. Gastronomica Culture 2, 46–62. 10.1525/gfc.2002.2.2.46

[ref8] Bell R. Pliner P. L. (2003). Time to eat: the relationship between the number of people eating and meal duration in three lunch settings. Appetite 41, 215–218. 10.1016/S0195-6663(03)00109-0, PMID: 14550324

[ref9] Bellisle F. Dalix A. M. (2001). Cognitive restraint can be offset by distraction, leading to increased meal intake in women. Am. J. Clin. Nutr. 74, 197–200. 10.1093/ajcn/74.2.197, PMID: 11470720

[ref10] Blass E. M. Anderson D. R. Kirkorian H. L. Pempek T. A. Price I. Koleini M. (2006). On the road to obesity: television viewing increases intake of high-density foods. Physiol. Behav. 88, 597–604. 10.1016/j.physbeh.2006.05.035, PMID: 16822530

[ref11] Boothby E. J. Clark M. S. Bargh J. A. (2014). Shared experiences are amplified. Psychol. Sci. 25, 2209–2216. 10.1177/0956797614551162, PMID: 25274583

[ref12] Boulos R. Vikre E. K. Oppenheimer S. Chang H. Kanarek R. B. (2012). ObesiTV: how television is influencing the obesity epidemic. Physiol. Behav. 107, 146–153. 10.1016/j.physbeh.2012.05.022 22677722

[ref13] Braude L. Stevenson R. J. (2014). Watching television while eating increases energy intake. Examining the mechanisms in female participants. Appetite 76, 9–16. 10.1016/j.appet.2014.01.005, PMID: 24462489

[ref14] Caldwell C. Hibbert S. A. (2002). The influence of music tempo and musical preference on restaurant patrons’ behavior. Psychol. Mark. 19, 895–917. 10.1002/mar.10043

[ref15] Cameron J. D. Maras D. Sigal R. J. Kenny G. P. Borghese M. M. Chaput J. P.. (2016). The mediating role of energy intake on the relationship between screen time behaviour and body mass index in adolescents with obesity: the HEARTY study. Appetite 107, 437–444. 10.1016/j.appet.2016.08.101, PMID: 27545672

[ref16] Cavazza N. Graziani A. R. Guidetti M. (2011). Looking for the right amount to eat at the restaurant: social influence effects when ordering. Soc. Influ. 6, 274–290. 10.1080/15534510.2011.632130

[ref17] Chapman C. Nilsson V. C. Thune H. Å. Cedernaes J. Le Grevès M. Hogenkamp P. S.. (2014). Watching TV and food intake: the role of content. PLoS One 9:e100602. 10.1371/journal.pone.0100602, PMID: 24983245 PMC4077693

[ref18] Choe H. (2019). Eating together multimodally: collaborative eating in *mukbang*, a Korean livestream of eating. Lang. Soc. 48, 171–208. 10.1017/S0047404518001355

[ref19] Clendenen V. I. Herman C. P. Polivy J. (1994). Social facilitation of eating among friends and strangers. Appetite 23, 1–13. 10.1006/appe.1994.1030, PMID: 7826053

[ref20] Coary S. Poor M. (2016). How consumer-generated images shape important consumption outcomes in the food domain. J. Consum. Mark. 33, 1–8. 10.1108/JCM-02-2015-1337

[ref21] Comber R. Barden P. Bryan-Kinns N. Olivier P. (2015). “Not sharing sushi: exploring social presence and connectedness at the telematic dinner party” in Eat, cook, grow: Mixing human-computer interactions with human-food interactions. eds. Choi J. H.-J. Foth M. Hearn G. (Cambridge, MA: MIT Press), 65–79.

[ref22] Comber R. Choi J. H.-J. Hoonhout J. O’Hara K. (2014). Designing for human-food interaction: an introduction to the special issue on ‘Food and interaction design’. Int. J. Hum. Comp. Stud. 72, 181–184. 10.1016/j.ijhcs.2013.09.001

[ref23] Conklin A. I. Forouhi N. G. Surtees P. Khaw K. T. Wareham N. J. Monsivais P. (2014). Social relationships and healthful dietary behaviour: evidence from over-50s in the EPIC cohort, UK. Soc. Sci. Med. 100, 167–175. 10.1016/j.socscimed.2013.08.018, PMID: 24035440 PMC3969105

[ref24] Coon K. Goldberg J. Rogers B. L. Tucker K. L. (2001). Relationships between use of television during meals and children’s food consumption patterns. Pediatrics 107:e7. 10.1542/peds.107.1.e7, PMID: 11134471

[ref25] Cruwys T. Bevelander K. E. Hermans R. C. (2015). Social modeling of eating: a review of when and why social influence affects food intake and choice. Appetite 86, 3–18. 10.1016/j.appet.2014.08.035, PMID: 25174571

[ref26] Cruwys T. Platow M. J. Angullia S. A. Chang J. M. Diler S. E. Kirchner J. L.. (2012). Modeling of food intake is moderated by salient psychological group membership. Appetite 58, 754–757. 10.1016/j.appet.2011.12.002, PMID: 22178007

[ref57] da Gonçalves R. F. M. de Barreto D. A. Monteiro P. I. Zangeronimo M. G. Castelo P. M. van der Bilt A.. (2019). Smartphone use while eating increases caloric ingestion. Physiol. Behav. 204, 93–99. 10.1016/j.physbeh.2019.02.021, PMID: 30776379

[ref27] Danesi G. (2012). Pleasures and stress of eating alone and eating together among French and German young adults. Menu 1, 77–91.

[ref28] de Castro J. M. (1990). Social facilitation of duration and size but not rate of the spontaneous meal intake in humans. Physiol. Behav. 47, 1129–1135. 10.1016/0031-9384(90)90363-9, PMID: 2395917

[ref29] de Castro J. M. (1994). Family and friends produce greater social facilitation of food intake than other companions. Physiol. Behav. 56, 445–455. 10.1016/0031-9384(94)90286-0, PMID: 7972393

[ref30] de Castro J. M. (2002). Age-related changes in the social, psychological, and temporal influences on food intake in free-living, healthy, adult humans. J. Gerontol. A Biol. 57, M368–M377. 10.1093/gerona/57.6.m368 12023266

[ref31] de Castro J. M. Brewer E. M. (1992). The amount eaten in meals by humans is a power function of the number of people present. Physiol. Behav. 51, 121–125. 10.1016/0031-9384(92)90212-K, PMID: 1741437

[ref32] de Castro J. M. Brewer E. M. Elmore D. K. Orozco S. (1990). Social facilitation of the spontaneous meal size of humans occurs regardless of time, place, alcohol or snacks. Appetite 15, 89–101. 10.1016/0195-6663(90)90042-7, PMID: 2268142

[ref33] Delistraty C. C. (2014). The importance of eating together: Family dinners build relationships, and help kids do better in school. *The Atlantic*, July 18th. Available at: https://www.theatlantic.com/health/archive/2014/07/the-importance-of-eating-together/374256/ (Accessed September 26, 2019).

[ref34] Donnar G. (2017). ‘Food porn’ or intimate sociality: committed celebrity and cultural performances of overeating in *meokbang*. Celebr. Stud. 8, 122–127. 10.1080/19392397.2016.1272857

[ref35] Douglas M. Nicod M. (1974). Taking the biscuit: the structure of British meals. New Society 30, 744–747.

[ref36] Dunbar R. I. M. (2017). Breaking bread: the functions of social eating. Adapt. Hum. Behav. Physiol. 3, 198–211. 10.1007/s40750-017-0061-4 PMC6979515 32025474

[ref37] Eleftheriou-Smith L.-M. (2017). VizEat: The app that lets you eat dinner in a stranger’s home. *The Independent*, April 10th. Available at: https://www.independent.co.uk/travel/europe/vizeat-app-eat-dinner-strangers-home-food-drink-local-hosts-paris-a7676441.html (Accessed September 26, 2019).

[ref38] Ensor J. (2013). Eat and then tweet, the modern way to dine out that’s driving chefs to distraction: Leading chefs have observed a rising number of customers taking photographs of their meals for social media profiles or blogs, to the irritation of other guests. *The Telegraph*, January 27th. Available at: http://www.telegraph.co.uk/foodanddrink/9828766/Eat-and-then-tweet-the-modern-way-to-dine-out-thats-driving-chefs-to-distraction.html (Accessed September 26, 2019).

[ref39] Feiler B. (2010). Should you Google at dinner? *The New York Times*, December 10th. Available at: https://www.nytimes.com/2010/12/12/fashion/12THISLIFE.html (Accessed September 26, 2019).

[ref40] Ferdman R. A. (2015). The most American thing there is: Eating alone. *The Washington Post*, August 18th. Available at: https://www.washingtonpost.com/news/wonk/wp/2015/08/18/eating-alone-is-a-fact-of-modern-american-life/?noredirect=on&utm_term=.8d87ff76cbd3 (Accessed September 26, 2019).

[ref41] Ferdous H. S. Ploderer B. Davis H. Vetere F. O’Hara K. (2016a). Commensality and the social use of technology during family mealtime. ACM Trans. Comp. Hum. Interac. 23, 37:1–37:26. 10.1145/2994146

[ref42] Ferdous H. S. Ploderer B. Davis H. Vetere F. O’Hara K. Farr-Wharton G. (2016b). “TableTalk: integrating personal devices and content for commensal experiences at the family dinner table” in Proceedings of the 2016 ACM international joint conference on pervasive and ubiquitous computing (New York, NY: ACM), 132–143.

[ref43] Ferdous H. S. Vetere F. Davis H. Ploderer B. O’Hara K. Comber R. (2017). “Celebratory technology to orchestrate the sharing of devices and stories during family mealtimes” in ACM CHI conference on human factors in computing systems (Denver, CO: ACM).

[ref44] Feunekes G. I. de Graaf C. van Staveren W. A. (1995). Social facilitation of food intake is mediated by meal duration. Physiol. Behav. 58, 551–558. 10.1016/0031-9384(95)00087-Y, PMID: 8587964

[ref45] Fischler C. (2011). Commensality, society and culture. Soc. Sci. Inf. 50, 528–548. 10.1177/0539018411413963

[ref46] Fishwick C. (2014). Table for one? Restaurant offers giant stuffed animals for company. *The Guardian*, May 6th. Available at: https://www.theguardian.com/world/2014/may/06/table-for-one-restaurant-giant-stuffed-animals-loneliness-japan (Accessed September 26, 2019).

[ref47] Fitzpatrick E. Edmunds L. Dennison B. (2007). Positive effects of family dinner are undone by television viewing. J. Am. Diet. Assoc. 107, 666–671. 10.1016/j.jada.2007.01.014, PMID: 17383273

[ref48] Foley-Fisher Z. Tsao V. Wang J. Fels S. (2010). NetPot: easy meal enjoyment for distant diners. Entertain. Comput. 6243, 446–448. 10.1007/978-3-642-15399-0_56

[ref49] Food Standards Agency (2016). Frequency of eating out or buying food to take away in the United Kingdom (UK) in 2016. *Statista - The Statistics Portal* Available at: https://www.statista.com/statistics/419297/eating-out-frequency-in-the-united-kingdom-uk/ (Accessed September 26, 2019).

[ref50] Fox J. Bailenson J. Binney J. (2009). Virtual experiences, physical behaviors: the effect of presence on imitation of an eating avatar. Presence Teleop. Virt. 18, 294–303. 10.1162/pres.18.4.294

[ref51] Freedom du Lac J. (2011). Solo diners find a new companion right at their fingertips. *The Washington Post*, October 28th. Available at: https://www.washingtonpost.com/local/solo-diners-find-a-new-companion-right-at-their-fingertips/2011/10/19/gIQAFPrCPM_story.html?utm_term=.0cec311cf240 (Accessed September 26, 2019).

[ref52] Frizzell N. (2016). Dinner for one – now that’s my kind of date. *Guardian*, April 14th. Available at: https://www.theguardian.com/commentisfree/2016/apr/13/dinner-for-one-date-solo-dining-eat?utm_source=esp&utm_medium=Email&utm_campaign=GU+Today+main+NEW+H&utm_term=167009&subid=16021322&CMP=EMCNEWEML6619I2 (Accessed September 26, 2019).

[ref53] Fulkerson J. A. Larson N. Horning M. Neumark-Sztainer D. (2014). A review of associations between family or shared meal frequency and dietary and weight status outcomes across the lifespan. J. Nutr. Educ. Behav. 46, 2–19. 10.1016/j.jneb.2013.07.012, PMID: 24054888

[ref54] Gardiner P. M. McCue K. D. Negash L. M. Cheng T. White L. F. Yinusa-Nyahkoon L.. (2017). Engaging women with an embodied conversational agent to deliver mindfulness and lifestyle recommendations: a feasibility randomized control trial. Patient Educ. Couns. 100, 1720–1729. 10.1016/j.pec.2017.04.015, PMID: 28495391 PMC5559098

[ref55] Goldfarb S. Tarver W. L. Sen B. (2014). Family structure and risk behaviors: the role of the family meal in assessing likelihood of adolescent risk behaviors. Psychol. Res. Behav. Manag. 7, 53–66. 10.2147/PRBM.S40461, PMID: 24627645 PMC3931580

[ref56] Goldman S. J. Herman C. P. Polivy J. (1991). Is the effect of a social model on eating attenuated by hunger? Appetite 17, 129–140. 10.1016/0195-6663(91)90068-4, PMID: 1763905

[ref58] Goodwin C. (1981). Conversational organization: Interaction between speakers and hearers. New York, NY: Academic Press.

[ref59] Gordon L. (2017). Single-person households will become a major consumption group. *Euromonitor International*, March 20th. Available at: https://blog.euromonitor.com/households-2030-singletons/ (Accessed September 26, 2019).

[ref60] Gore S. A. Foster J. A. DiLillo V. G. Kirk K. Smith West D. (2003). Television viewing and snacking. Eat. Behav. 4, 399–405. 10.1016/S1471-0153(03)00053-9, PMID: 15000965

[ref61] Grevet C. Tang A. Mynatt E. (2012). “Eating alone, together: new forms of commensality” in Proceedings of the 17th ACM international conference on supporting group work (New York, NY: ACM), 103–106.

[ref62] Grimes A. Harper R. (2008). “Celebratory technology: new directions for food research in HCI” in Proceedings of the SIGCHI conference on human factors in computing systems (New York, NY: ACM), 467–476.

[ref63] Hammons A. Fiese B. H. (2011). Is frequency of shared family meals related to the nutritional health of children and adolescents? A metaanalysis. Pediatrics 127, e1565–e1574. 10.1542/peds.2010-1440 21536618 PMC3387875

[ref64] Hawkley L. C. Cacioppo J. T. (2010). Loneliness matters: a theoretical and empirical review of consequences and mechanisms. Ann. Behav. Med. 40, 218–227. 10.1007/s12160-010-9210-8, PMID: 20652462 PMC3874845

[ref65] Heidrich F. Kasugai K. Röcker C. Russell P. Ziefle M. (2012). “RoomXT: advanced video communication for joint dining over a distance” in 6^th^ international conference on pervasive computing Technologies for Healthcare (PervasiveHealth) and workshops 2012 (New York, NY: IEEE), 211–214.

[ref66] Herman C. P. (2015). The social facilitation of eating. A review. Appetite 86, 61–73. 10.1016/j.appet.2014.09.016, PMID: 25265153

[ref67] Herman C. P. (2017). The social facilitation of eating or the facilitation of social eating? J. Eat. Disord. 5:16. 10.1186/s40337-017-0146-2 28451432 PMC5406877

[ref68] Herman C. P. Roth D. A. Polivy J. (2003). Effects of the presence of others on food intake: a normative interpretation. Psychol. Bull. 129, 873–886. 10.1037/0033-2909.129.6.873, PMID: 14599286

[ref69] Hermans R. C. Engels R. C. Larsen J. K. Herman C. P. (2009). Modeling of palatable food intake. The influence of quality of social interaction. Appetite 52, 801–804. 10.1016/j.appet.2009.03.008, PMID: 19501786

[ref70] Hermans R. C. J. Hermsen S. Robinson E. Higgs S. Mars M. Frost J. H. (2017). The effect of real-time vibrotactile feedback delivered through an augmented fork on eating rate, satiation, and food intake. Appetite 113, 7–13. 10.1016/j.appet.2017.02.014, PMID: 28192220

[ref71] Hermans R. C. Lichtwarck-Aschoff A. Bevelander K. E. Herman C. P. Larsen J. K. Engels R. C. (2012). Mimicry of food intake: the dynamic interplay between eating companions. PLoS One 7:e31027. 10.1371/journal.pone.0031027, PMID: 22312438 PMC3270030

[ref72] Hermsen S. Frost J. H. Robinson E. Higgs S. Mars M. Hermans R. C. (2016). Evaluation of a smart fork to decelerate eating rate. J. Acad. Nutr. Diet. 116, 1066–1067. 10.1016/j.jand.2015.11.004, PMID: 26785908

[ref73] Hetherington M. M. Anderson A. S. Norton G. N. Newson L. (2006). Situational effects on meal intake: a comparison of eating alone and eating with others. Physiol. Behav. 88, 498–505. 10.1016/j.physbeh.2006.04.025, PMID: 16757007

[ref74] Higgs S. Thomas J. (2016). Social influences on eating. Curr. Opin. Behav. Sci. 9, 1–6. 10.1016/j.cobeha.2015.10.005

[ref75] Hiniker A. Schoenebeck S. Y. Kientz J. A. (2016). “Not at the dinner table: Parents’ and children’s perspectives on family technology rules” in Proceedings of the 19^th^ ACM conference on computer-supported cooperative work & social computing (New York, NY: ACM), 1376–1389.

[ref76] Hirsch E. S. Kramer E. M. (1993). “Situational influences on food intake” in Nutritional needs in hot environments. ed. Marriott B. M. (Washington DC: National Academy Press), 215–243.

[ref77] Horowitz B. (2010). Will robots help the elderly live at home longer? *Scientific American*, June 21st. Available at: https://www.scientificamerican.com/article/robot-elder-care/?redirect=1 (Accessed September 26, 2019).

[ref78] Hurst G. (2018). Eating meals alone is biggest lifestyle cause of unhappiness. *The Times*, May 22nd, 15.

[ref79] Johnston J. P. (1977). A hundred years eating: Food, drink and the daily diet in Britain since the late nineteenth century. Dublin, IE: Gill & Macmillan.

[ref80] Jones M. (2008). Feast: Why humans share food. Oxford, UK: Oxford University Press.

[ref81] Jonsson I. Pipping Ekström M. (2009). “Gender perspective on the solo dinner [sic.] as restaurant customer” in Meals in science and practice: Interdisciplinary research and business applications. ed. Meiselman H. (Cambridge, UK: CRC Press & Woodhead), 236–249.

[ref82] Kim Y. (2018). “Sell your loneliness: Mukbang culture and multisensorial capitalism in South Korea” in Routledge handbook of cultural and creative industries in Asia. eds. Lim L. Lee H.-K. (London, UK: Routledge), 225–238.

[ref83] King S. C. Weber A. J. Meiselman H. L. Lv N. (2004). The effect of meal situation, social interaction, physical environment and choice on food acceptability. Food Qual. Prefer. 15, 645–653. 10.1016/j.foodqual.2004.04.010

[ref84] Klesges R. C. Bartsch D. Norwood J. D. Kautzman D. Haugrud S. (2006). The effects of selected social variables on the eating behaviour of adults in the natural environments. Int. J. Eat. Disord. 3, 35–41. 10.1002/1098-108X(198422)3:4<35::AID-EAT2260030405>3.0.CO;2-7

[ref85] Klinenberg E. (2012). I want to be alone: The rise and rise of solo living. *The Guardian*, March 30th. Available at: https://www.theguardian.com/lifeandstyle/2012/mar/30/the-rise-of-solo-living (Accessed September 26, 2019).

[ref86] Klinenberg E. (2013). Going solo: The extraordinary rise and surprising appeal of living alone. New York, NY: Penguin.

[ref87] Kononova A. McAlister A. Oh H. J. (2018). Screen overload: pleasant multitasking with screen devices leads to the choice of healthful over less healthful snacks when compared with unpleasant multitasking. Comput. Hum. Behav. 80, 1–11. 10.1016/j.chb.2017.10.042

[ref88] Lanza J. (2004). Elevator music: A surreal history of Muzak, easy-listening, and other moodsong. Ann Arbor, MI: University of Michigan Press.

[ref89] Larson N. I. Nelson M. C. Neumark-Sztainer D. Story M. Hannan P. J. (2009). Making time for meals. Meal structure and associations with dietary intake in young adults. J. Am. Diet. Assoc. 109, 72–79. 10.1016/j.jada.2008.10.017, PMID: 19103325

[ref90] Laurier E. Wiggins S. (2011). Finishing the family meal. The interactional organisation of satiety. Appetite 56, 53–64. 10.1016/j.appet.2010.11.138, PMID: 21095211

[ref91] Levine A. S. (2016). New York today: Where to eat alone. *The New York Times*, February 11th. Available at: http://www.nytimes.com/2016/02/11/nyregion/new-york-today-where-to-eat-alone.html?_r=0 (Accessed September 26, 2019).

[ref92] Lifshitz F. Lifshitz J. Z. (2014). Globesity: the root causes of the obesity epidemic in the USA and now worldwide. Pediatr. Endocrinol. Rev. 12, 17–34. 25345082

[ref93] Luckhurst P. (2015). Table for one? *Evening Standard*, October 21st.

[ref94] Mäkelä J. (2009). “Meals: the social perspective” in Meals in science and practice: Interdisciplinary research and business applications. ed. Meiselman H. L. (Cambridge, UK: CRC Press & Woodhead), 37–49.

[ref95] Marsh S. Mhurchu C. N. Jiang Y. Maddison R. (2015). Modern screen-use behaviors: the effects of single-and multi-screen use on energy intake. J. Adolesc. Health 56, 543–549. 10.1016/j.jadohealth.2015.01.009, PMID: 25772065

[ref96] Marsh S. Mhurchu C. N. Maddison R. (2013). The non-advertising effects of screen-based sedentary activities on acute eating behaviours in children, adolescents, and young adults. A systematic review. Appetite 71, 259–273. 10.1016/j.appet.2013.08.017, PMID: 24001394

[ref97] Marshall J. A. Lopez T. K. Shetterly S. M. Morgenstern N. E. Baer K. Swenson C.. (1999). Indicators of nutritional risk in a rural elderly Hispanic and non-Hispanic white population: San Luis Valley health and aging study. J. Am. Diet. Assoc. 99, 315–322. 10.1016/S0002-8223(99)00081-4, PMID: 10076583

[ref98] Marx P. (2018). Learning to love robots. *The New Yorker*, November 26th. Available at: https://www.newyorker.com/magazine/2018/11/26/learning-to-love-robots (Accessed September 26, 2019).

[ref99] Mathur U. Stevenson R. J. (2015). Television and eating: repetition enhances food intake. Front. Psychol. 6:1657. 10.3389/fpsyg.2015.01657, PMID: 26579040 PMC4630539

[ref100] McColl D. Nejat G. (2013). Meal-time with a socially assistive robot and older adults at a long-term care facility. J. Hum. Robot Interac. 2, 152–171. 10.5898/JHRI.2.1.McColl

[ref101] McElrea H. Standing L. (1992). Fast music causes fast drinking. Percept. Mot. Skills 75:362. 1408589 10.2466/pms.1992.75.2.362

[ref102] McFerran B. Dahl D. W. Fitzsimons G. J. Morales A. C. (2009). I’ll have what she’s having: effects of social influence and body type on the food choices of others. J. Consum. Res. 36, 915–929. 10.1086/644611

[ref103] McIntosh A. (1999). “The family meal and its significance in global times” in Food in global history. ed. Grew R. (Boulder, CO: Westview Press), 217–239.

[ref104] Mestdag I. (2005). Disappearance of the traditional meal: temporal, social and spatial destructuration. Appetite 45, 62–74. 10.1016/j.appet.2005.03.003, PMID: 15896879

[ref105] Mondada L. (2009). The methodical organization of talking and eating: assessments in dinner conversations. Food Quality Prefer. 20, 558–571. 10.1016/j.foodqual.2009.03.006

[ref106] Moser C. Schoenebeck S. Y. Reinecke K. (2016). “Technology at the table: attitudes about mobile phone use at mealtimes” in *Proceedings of the 2016 CHI Conference on Human Factors in Computing Systems*. New York, NY: ACM, 1881–1892.

[ref107] Muhammad L. (2012). More workers work through lunch or eat at their desks. *USA Today*, April 13th. Available at: http://usatoday30.usatoday.com/money/workplace/story/2012-04-15/lunch-at-work/54167808/1 (Accessed September 26, 2019).

[ref108] Munro N. D. Grosman L. (2010). Early evidence (ca. 12,000 B.P.) for feasting at a burial cave in Israel. Proc. Natl. Acad. Sci. USA 107, 15362–15366. 10.1073/pnas.1001809107, PMID: 20805510 PMC2932561

[ref109] Murcott A. (1997). “Family meals – a thing of the past?” in Food, health and nutrition. ed. Caplan P. (London, UK: Routledge), 32–49.

[ref110] Muston S. (2015). The blissful silence of a peaceful meal for one. *The Independent*, January 16th. Available at: http://www.independent.co.uk/life-style/food-and-drink/features/the-blissful-silence-of-a-peaceful-meal-for-one-9981463.html (Accessed September 26, 2019).

[ref111] Nakata R. Kawai N. (2017). The “social” facilitation of eating without the presence of others: self-reflection on eating makes food taste better and people eat more. Physiol. Behav. 179, 23–29. 10.1016/j.physbeh.2017.05.022, PMID: 28528894

[ref112] Narumi T. Ban Y. Kajinami T. Tanikawa T. Hirose M. (2012). “Augmented perception of satiety: controlling food consumption by changing apparent size of food with augmented reality” in Proceedings 2012 ACM annual conference human factors in computing systems; CHI 2012, may 5–10, 2012, Austin, TX. New York, NY: ACM Press.

[ref113] National Center for Health Statistics (2014). *Health, United States, 2013: With Special Feature on Prescription Drugs*. Hyattsville, MD. 24967476

[ref114] Nawahdah M. Inoue T. (2013). “Virtually dining together in time-shifted environment: KIZUNA design” in *CSCW ‘13, Proceedings of the 2013 Conference on Computer Supported Work*, San Antonio, TX. New York, NY: ACM, 779–788.

[ref115] Neumark-Sztainer D. Hannan P. J. Story M. Croll J. Perry C. (2003). Family meal patterns: associations with sociodemographic characteristics and improved dietary intake among adolescents. J. Am. Diet. Assoc. 103, 317–322. 10.1053/jada.2003.50048, PMID: 12616252

[ref116] NPD Group (2018). Share of dinners that are consumed inside and outside of the home in the United States as of July 2018. *Statista - The Statistics Portal* Available at: https://www.statista.com/statistics/967505/eating-in-or-out-dining-preferences-us/ (Accessed September 26, 2019).

[ref117] O’Hara K. Helmes J. Sellen A. Harper R. ten Bhömer M. van den Hoven E. (2012). Food for talk: Phototalk in the context of sharing a meal. Hum. Comp. Interac. 27, 124–150. 10.1080/07370024.2012.656069

[ref118] Obrist M. Tu Y. Yao L. Velasco C. (2019). Space food experiences: designing passenger’s eating experiences for future space travel scenarios. Front. Comp. Sci. 1:3. 10.3389/fcomp.2019.00003

[ref119] Ochs E. Pontecorvo C. Fasulo A. (1996). Socializing taste. Ethnos 61, 7–46.

[ref120] Ochs E. Shohet M. (2006). “The cultural structuring of mealtime socialization” in Family mealtime as a context of development and socialization. eds. Larson R. Wiley A. Branscomb K. (San Francisco, CA: Jossey-Bass), 35–50. 10.1002/cd.154 16646498

[ref121] Oldham-Cooper R. E. Hardman C. A. Nicoll C. E. Rogers P. J. Brunstrom J. M. (2011). Playing a computer game during lunch affects fullness, memory for lunch, and later snack intake. Am. J. Clin. Nutr. 93, 308–313. 10.3945/ajcn.110.004580, PMID: 21147857

[ref122] OpenTable (2015). You’re not alone: OpenTable study reveals rise in solo dining, names top restaurants for solo diners. *OpenTable*, October 7th. Available at: https://blog.opentable.com/2015/youre-not-alone-opentable-study-reveals-rise-in-solo-dining-names-top-restaurants-for-solo-diners/ (Accessed September 26, 2019).

[ref123] Osborn C. L. Marshall M. (1992). Promoting meal-time independence. Geriatr. Nurs. 13, 254–256. 10.1016/S0197-4572(05)80414-8, PMID: 1327987

[ref124] Palmer S. (2006). Toxic childhood. London, UK: Orion Books.

[ref125] Parra M. O. Favela J. Castro L. A. Morales A. (2018). Monitoring eating behaviors for a nutritionist E-assistant using crowdsourcing. Computer 51, 43–51. 10.1109/MC.2018.1731078

[ref126] Pavia W. (2019). Table for one? New York restaurants welcome era of solo diners. *The Times*, February 15th, 35.

[ref127] Pellegrino R. Luckett C. R. Shinn S. E. Mayfield S. Gude K. Rhea A. (2015). Effects of background sound on consumers’ sensory discriminatory ability among foods. Food Quality Prefer. 43, 71–78. 10.1016/j.foodqual.2015.02.014

[ref128] Pereira B. Sung B. Lee S. (2019). I like watching other people eat: a cross-cultural analysis of the antecedents of attitudes towards Mukbang. Australas. Mark. J. 27, 78–90. 10.1016/j.ausmj.2019.03.001

[ref129] Pesce N. I. (2016). Facebook launches new food delivery service, continues to colonize your life. *New York Daily News*, October 21st. Available at: http://www.nydailynews.com/life-style/facebook-launches-new-food-delivery-service-article-1.28396 not available in Europe currently. (Accessed September 26, 2019).

[ref130] Phua J. Jin S. V. Kim J. J. (2017). Uses and gratifications of social networking sites for bridging and bonding social capital: a comparison of Facebook, twitter, Instagram, and Snapchat. Comput. Hum. Behav. 72, 115–122. 10.1016/j.chb.2017.02.041

[ref131] Pliner P. Bell R. (2009). “A table for one: the pain and pleasure of eating alone” in Meals in science and practice: Interdisciplinary research and business applications. ed. Meiselman H. L. (Cambridge, UK: Woodhead Publishing Limited), 169–189.

[ref132] Pliner P. Mann P. (2004). Influence of social norms and palatability on amount consumed and food choice. Appetite 42, 227–237. 10.1016/j.appet.2003.12.001, PMID: 15010187

[ref133] Polivy J. (2017). What’s that you’re eating? Social comparison and eating behavior. J. Eat. Disord. 5:18. 10.1186/s40337-017-0148-0 28465828 PMC5408479

[ref134] Polivy J. Pliner P. (2015). “She got more than me”. Social comparison and the social context of eating. Appetite 86, 88–95. 10.1016/j.appet.2014.08.007, PMID: 25128833

[ref135] Poor M. Duhachek A. Krishnan H. S. (2013). How images of other consumers influence subsequent taste perceptions. J. Mark. 77, 124–139. 10.1509/jm.12.0021

[ref136] Poulain J.-P. (2002). The contemporary diet in France: “Destructuration” or from commensalism to “vagabond feeding”. Appetite 39, 43–55. 10.1006/appe.2001.0461, PMID: 12160564

[ref137] Quested T. Luzecka P. (2014). Household food and drink waste: A people focus. *Waste & Resources Action Programme*, CFP204. Available at: http://www.wrap.org.uk/sites/files/wrap/People-focused%20report%20v6_5%20full.pdf (Accessed September 26, 2019).

[ref138] Quigley K. K. Hermann W. D. Warde W. D. (2008). Nutritional risk among Oklahoma congregate meal participants. J. Nutr. Educ. Behav. 40, 89–93. 10.1016/j.jneb.2007.08.014, PMID: 18314084

[ref139] Radesky J. S. Kistin C. J. Zuckerman B. Nitzberg K. Gross J. Kaplan-Sanoff M.. (2014). Patterns of mobile device use by caregivers and children during meals in fast food restaurants. Pediatrics 133, e843–e849. 10.1542/peds.2013-3703, PMID: 24616357

[ref140] Randall N. Joshi S. Liu X. (2018). “Health-e-eater: dinnertime companion robot and magic plate for improving eating habits in children from low-income families” in Companion of the 2018 ACM/IEEE international conference on human-robot interaction. New York, NY: ACM, 361–362.

[ref141] Ratner R. K. Hamilton R. W. (2015). Inhibited from bowling alone. J. Consum. Res. 42, 266–283. 10.1093/jcr/ucv012

[ref142] Rimer S. (2009). Play with your food, just don’t text! *The New York Times*, May 26th. Available at: https://www.nytimes.com/2009/05/27/dining/27text.html?pagewanted=all (Accessed September 26, 2019).

[ref143] Ritschel H. Seiderer A. Janowski K. Aslan I. André E. (2018). “Drink-O-mender: an adaptive robotic drink advisor” in *Proceedings of the 3^rd^ International Workshop on Multisensory Approaches to Human-Food Interaction 2018; 20^th^ ACM International Conference on Multimodal Interactions - ICMI ‘18* (article 3). October 16th, Boulder, CO (New York, NY: ACM Press).

[ref144] Roballey T. C. McGreevy C. Rongo R. R. Schwantes M. L. Steger P. J. Wininger M. A. (1985). The effect of music on eating behavior. Bull. Psychon. Soc. 23, 221–222. 10.3758/BF03329832

[ref145] Robinson E. Aveyard P. Daley A. Jolly K. Lewis A. Lycett D.. (2013). Eating attentively: a systematic review and meta-analysis of the effect of food intake memory and awareness on eating. Am. J. Clin. Nutr. 97, 728–742. 10.3945/ajcn.112.045245, PMID: 23446890 PMC3607652

[ref146] Rotenberg R. (1981). The impact of industrialization on meal patterns in Vienna, Austria. Ecol. Food Nutr. 11, 25–35.

[ref147] Rotondi V. Stanca L. Tomasuolo M. (2017). Connecting alone: smartphone use, quality of social interactions and well-being. J. Econ. Psychol. 63, 17–26. 10.1016/j.joep.2017.09.001

[ref148] Rumbelow H. (2015). Tired of takeaways? Try supper in a stranger’s home with the Airbnb of dining. *The Times*, November 19th (Times2), 6–7.

[ref149] Salvy S. J. Jarrin D. Paluch R. Irfan N. Pliner P. (2007). Effects of social influence on eating in couples, friends and strangers. Appetite 49, 92–99. 10.1016/j.appet.2006.12.004, PMID: 17296248

[ref150] Sanghani R. (2014). ‘Table for one, please’: Would you ever dine out alone at night in Britain? *The Daily Telegraph*, April 25th. Available at: https://www.telegraph.co.uk/women/womens-life/10787740/Table-for-one-please-would-you-ever-dine-out-alone-at-night-in-Britain.html (Accessed September 26, 2019).

[ref151] Schell E. S. Kayser-Jones J. (1999). The effect of role-taking on caregiver-resident meal-time interaction. Appl. Nurs. Res. 12, 38–44. 10.1016/S0897-1897(99)80167-0, PMID: 10048240

[ref152] Seddon L. Berry N. (1996). Media-induced disinhibition of dietary restraint. Br. J. Health Psychol. 1, 27–33. 10.1111/j.2044-8287.1996.tb00489.x

[ref153] Severson K. (2016). It’s dinner in a box. But are meal delivery kits cooking? *The New York Times*, April 4th. Available at: https://www.nytimes.com/2016/04/06/dining/meal-delivery-service-subscription-boxes.html (Accessed September 26, 2019).

[ref154] Simnel G. (1910/1994). Sociology of the meal, trans. M. Symons. Food Foodways 5, 345–350.

[ref155] Sobal J. (2000). “Sociability and the meal: facilitation, commensality, and interaction” in Dimensions of the meal: The science, culture, business, and art of eating. ed. Meiselman H. (Gaithersburg, MD: Aspen), 119–133.

[ref156] Sobal J. Hanson K. (2011). Family meals and body weight in US adults. Public Health Nutr. 14, 1555–1562. 10.1017/S1368980011000127, PMID: 21356147

[ref157] Sobal J. Nelson M. K. (2003). Commensal eating patterns. A community study. Appetite 41, 181–190. 10.1016/S0195-6663(03)00078-3, PMID: 14550316

[ref158] Spang R. L. (2000). The invention of the restaurant: Paris and modern gastronomic culture. Cambridge, MA: Harvard University Press.

[ref159] Spence C. (2016). Gastrodiplomacy: assessing the role of food in decision-making. Flavour 5:4. 10.1186/s13411-016-0050-8

[ref160] Spence C. (2017a). Gastrophysics: The new science of eating. London, UK: Viking Penguin.

[ref161] Spence C. (2017b). Hospital food. Flavour 6:3. 10.1186/s13411-017-0055-y

[ref162] Spence C. (2017c). “Sonic seasoning” in Audio branding: Using sound to build your brand. eds. Minsky L. Fahey C. (London, UK: Kogan Page), 52–58.

[ref163] Spence C. (2018). “Mirror, mirror on the wall”: can visual illusions be used to ‘trick’ people into eating less? Int. J. Gastr. Food Sci. 11, 31–34. 10.1016/j.ijgfs.2017.11.002

[ref164] Spence C. Okajima K. Cheok A. D. Petit O. Michel C. (2016). Eating with our eyes: from visual hunger to digital satiation. Brain Cogn. 110, 53–63. 10.1016/j.bandc.2015.08.006, PMID: 26432045

[ref165] Spence C. Piqueras-Fiszman B. (2013). Technology at the dining table. Flavour 2:16. 10.1186/2044-7248-2-16

[ref166] Spence C. Piqueras-Fiszman B. (2014). The perfect meal: The multisensory science of food and dining. Oxford, UK: Wiley-Blackwell.

[ref1470] Spence C. Reinoso-Carvalho F. Velasco C. Wang Q. J. (2019). Extrinsic auditory contributions to food perception & consumer behaviour: An interdisciplinary review. Multisens. Res. 32, 275–318. 31059484 10.1163/22134808-20191403

[ref167] Statista (2016). Frequency of eating out or buying food to take away in the United Kingdom (UK) in 2016. *Statista – The Statistics Portal* Available at: https://www.statista.com/statistics/419297/eating-out-frequency-in-the-united-kingdom-uk/ (Accessed September 26, 2019).

[ref168] Statista (2017). Share of consumers using food delivery services in the United States in 2016, by number of household members. *Statista - The Statistics Portal* Available at: https://www.statista.com/statistics/650544/frequency-of-using-food-delivery-services-us-by-household-members/ (Accessed September 26, 2019).

[ref169] Statista (2018). Number of users forecast for the Online Food Delivery market in Europe from 2017 to 2023 (in million). *Statista - The Statistics Portal* Available at: https://www.statista.com/statistics/696539/online-food-delivery-users-by-segment-in-europe/ (Accessed September 26, 2019).

[ref170] Statista (2019a). Restaurant-to-consumer delivery. *Statista - The Statistics Portal* Available at: https://www.statista.com/outlook/375/100/restaurant-to-consumer-delivery/worldwide#market-revenue (Accessed September 26, 2019).

[ref171] Statista (2019b). Eating on the run among U.S. consumers in 2018, by generation. *Statista - The Statistics Portal* Available at: https://www.statista.com/statistics/921133/eating-habits-of-us-consumers-by-generation/ (Accessed September 26, 2019).

[ref172] Statista Survey (2016). Which of these statements about ordering food for delivery apply to you? *Statista - The Statistics Portal* Available at: https://www.statista.com/statistics/668293/reasons-consumers-order-food-for-delivery-us/ (Accessed September 26, 2019).

[ref173] Steel C. (2008). Hungry city: How food shapes our lives. London, UK: Chatto & Windus.

[ref174] Strahan E. J. Spencer S. J. Zanna M. P. (2007). Don’t take another bite. How sociocultural norms for appearance affect women’s eating behavior. Body Image 4, 331–342. 10.1016/j.bodyim.2007.06.003, PMID: 18089279

[ref176] Tamir D. I. Templeton E. M. Ward A. F. Zakid J. (2018). Media usage diminishes memory for experiences. J. Exp. Soc. Psychol. 76, 161–168. 10.1016/j.jesp.2018.01.006

[ref177] Tani Y. Kondo N. Takagi D. Saito M. Hikichi H. Ojima T. (2015a). Combined effects of eating alone and living alone on unhealthy dietary behaviors, obesity and underweight in older Japanese adults: results of the JAGES. Appetite 95, 1–8. 10.1016/j.appet.2015.06.005 26116391

[ref178] Tani Y. Sasaki Y. Haseda M. Kondo K. Kondo N. (2015b). Eating alone and depression in older men and women by cohabitation status: the JAGES longitudinal survey. Age Ageing 44, 1019–1026. 10.1093/ageing/afv145 26504120 PMC4621239

[ref179] Torres C. C. McIntosh W. A. Kubena K. S. (1992). Social network and social background characteristics of elderly who live alone and eat alone. J. Aging Health 32, 365–373.

[ref180] Troisi J. D. Gabriel S. Derrick J. L. Geisler A. (2015). Threatened belonging and preference for comfort food among the securely attached. Appetite 90, 58–64. 10.1016/j.appet.2015.02.029, PMID: 25728881

[ref181] Tsujita H. Yarosh S. Abowd G. (2010). CU-later: A communication system considering time difference. *UbiComp’10*, September 26–29, Copenhagen, Denmark. New York, NY: ACM. 978–1–60558-843-8/10/09.

[ref182] US Census Bureau (2018a). Number of single-person households in the U.S. from 1960 to 2017 (in millions). *Statista - The Statistics Portal* Available at: https://www.statista.com/statistics/242022/number-of-single-person-households-in-the-us/ (Accessed September 26, 2019).

[ref183] US Census Bureau (2018b). U.S. Census Bureau Releases 2018 Families and Living Arrangements Tables. *US Census Bureau*, November 14th. Available at: https://www.census.gov/newsroom/press-releases/2018/families.html (Accessed September 26, 2019).

[ref184] van der Zee T. Anaya J. Brown N. J. L. (2017). Statistical heartburn: an attempt to digest four pizza publications from the Cornell food and brand lab. BMC Nutr. 3:54. 10.1186/s40795-017-0167-x PMC7050813 32153834

[ref185] Vice Food (2015). The food porn superstars of South Korea: Mukbang. *Munchies.* Available at: http://munchies.vice.com/videos/munchies-presents-mukbang (Accessed September 26, 2019).

[ref186] Victor A. (2015). Table for one, please! Number of solo diners DOUBLES in two years as eating alone is viewed as liberating rather than a lonely experience. *Daily Mail Online*, July 13th. Available at: http://www.dailymail.co.uk/femail/food/article-3156420/OpenTable-study-reveals-number-solo-diners-DOUBLES-two-years.html (Accessed September 26, 2019).

[ref187] Wei J. Wang X. Peiris R. L. Choi Y. Martinez X. R. Tache R. (2011). “CoDine: an interactive multi-sensory system for remote dining” in Proceedings of the 13th international conference on ubiquitous computing. (New York, NY: ACM), 21–30.

[ref188] Winsight Grocery Business (2018). Eating on the run among U.S. consumers in 2018, by generation. *Statista - The Statistics Portal* Available at: https://www.statista.com/statistics/921133/eating-habits-of-us-consumers-by-generation/ (Accessed September 26, 2019).

[ref189] Woolley K. Fishbach A. (2017). A recipe for friendship: similar food consumption promotes trust and cooperation. J. Consum. Psychol. 27, 1–10. 10.1016/j.jcps.2016.06.003

[ref190] Wright L. Hickson M. Frost G. (2006). Eating together is important: using a dining room in an acute elderly medical ward increases energy intake. J. Hum. Nutr. Diet. 19, 23–26. 10.1111/j.1365-277X.2006.00658.x, PMID: 16448471

[ref191] Young M. E. Mizzau M. Mai N. T. Sirisegaram A. Wilson M. (2009). Food for thought. What you eat depends on your sex and eating companions. Appetite 53, 268–271. 10.1016/j.appet.2009.07.021, PMID: 19646494

[ref192] Zhou S. Shapiro M. A. Wansink B. (2017). The audience eats more if a movie character keeps eating: an unconscious mechanism for media influence on eating behaviors. Appetite 108, 407–415. 10.1016/j.appet.2016.10.028, PMID: 27780785

